# Identification and Validation of Potential Biomarkers and Their Functions in Acute Kidney Injury

**DOI:** 10.3389/fgene.2020.00411

**Published:** 2020-05-12

**Authors:** Jianwen Chen, Yalei Chen, Alberto Olivero, Xiangmei Chen

**Affiliations:** ^1^Department of Nephrology, Chinese PLA General Hospital, Chinese PLA Institute of Nephrology, Beijing Key Laboratory of Kidney Disease, State Key Laboratory of Kidney Diseases, National Clinical Research Center for Kidney Diseases, Chinese People’s Liberation Army General Hospital, Beijing, China; ^2^Department of Critical Care Medicine, Beijing Electric Power Hospital, Beijing, China; ^3^Department of Urology, San Martino Policlinico Hospital, University of Genoa, Genoa, Italy

**Keywords:** acute kidney injury, microarray, high-throughput sequencing, integrated analysis, differentially expressed gene, interaction network analysis, validation

## Abstract

Acute kidney injury (AKI) is a global public health concern associated with high morbidity, mortality, and health-care costs, and the therapeutic measures are still limited. This study aims to investigate crucial genes correlated with AKI, and their potential functions, which might contribute to a better understanding of AKI pathogenesis. The high-throughput data GSE52004 and GSE98622 were downloaded from Gene Expression Omnibus; four group sets were extracted and integrated. Differentially expressed genes (DEGs) in the four group sets were identified by limma package in R software. The overlapping DEGs among four group sets were further analyzed by the VennDiagram package, and their potential functions were analyzed by the GO and KEGG pathway enrichment analyses using the DAVID database. Furthermore, the protein–protein interaction (PPI) network was constructed by STRING, and the functional modules of the PPI network were filtered by MCODE and ClusterOne in Cytoscape. Hub genes of overlapping DEGs were identified by Cyto-Hubba and cytoNCA. The expression of 35 key genes was validated by quantitative real-time PCR (qRT-PCR). Western blot and immunofluorescence were performed to validate an important gene Egr1. A total of 722 overlapping DEGs were differentially expressed in at least three group sets. These genes mainly enriched in cell proliferation and fibroblast proliferation. Additionally, 5 significant modules and 21 hub genes, such as Havcr1, Krt20, Sox9, Egr1, Timp1, Serpine1, Edn1, and Apln were screened by analyzing the PPI networks. The 5 significant modules were mainly enriched in complement and coagulation cascades and Metabolic pathways, and the top 21 hub genes were mainly enriched in positive regulation of cell proliferation. Through validation, Krt20 were identified as the top 1 upregulated genes with a log2 (fold change) larger than 10 in all these 35 genes, and 21 genes were validated as significantly upregulated; Egr1 was validated as an upregulated gene in AKI in both RNA and protein level. In conclusion, by integrated analysis of different high-throughput data and validation by experiment, several crucial genes were identified in AKI, such as Havcr1, Krt20, Sox9, Egr1, Timp1, Serpine1, Edn1, and Apln. These genes were very important in the process of AKI, which could be further utilized to explore novel diagnostic and therapeutic strategies.

## Introduction

Acute kidney injury (AKI) is a syndrome characterized by the rapid loss of the kidney’s excretory function and is typically diagnosed by the decreased urine output or accumulation of end products of nitrogen metabolism (urea and creatinine), or both (Bellomo et al., 2012). AKI is a global public health concern associated with high morbidity, mortality, and health-care costs (Zuk and Bonventre, 2016). It occurs in about 13.3 million people per year and is thought to contribute to about 1.7 million deaths every year (Mehta et al., 2015). Data show that 21% of hospital admissions were affected with AKI at least one time, and patients who require dialysis or those with Kidney Disease: Improving Global Outcomes (KDIGO) stage 3 had a high mortality rate (42 and 46%) (Mehta et al., 2015). About 25.7% of AKI hospitalized patients are still unable to recover after active treatment, and even if postdischarge kidney function returns to normal, AKI patients are still at substantially increased risk of developing chronic kidney disease (CKD) (Sawhney et al., 2017).

Despite extensive investigation of AKI in experimental models, the clinical outcome of AKI has not been improved substantially in the last decade (Liu et al., 2017), and the therapeutic measures for AKI are still limited; no therapeutic interventions except dialysis can reliably improve the survival outcome, limit injury, or speed recovery (Bellomo et al., 2012). In recent decades, the experimental mouse ischemia–reperfusion injury (IRI) model of AKI has been widely applied to study the pathogenesis and injury outcome of ischemic AKI (Hu et al., 2013), while the underlying mechanisms of AKI remain largely unclear.

Microarray and high-throughput sequencing have been widely used to explore the mechanisms of a series of diseases (Zhang et al., 2017). At present, a great number of studies have been performed on AKI gene expression profiles (Wei et al., 2010; Liu et al., 2014; Cui et al., 2016; Liu et al., 2017), and these studies have screened many differentially expressed genes (DEGs) that may be involved in the pathogenesis and progression of AKI. However, their results of DEGs are often not the same due to the heterogeneity among each independent experiment. Thus, single-cohort studies still have many limitations, and the integrated analysis of high-throughput data from different gene expression profiles may solve these difficult problems and enable the discovery of some reliable and effective diagnosis and therapeutic molecular markers (Yang et al., 2018).

In the present study, we aimed to obtain deep insights into the mechanisms and find some potential molecular markers for prognosis and early detection, as well as therapeutic drug targets for treating AKI. We perform an integrated analysis of two datasets GSE52004 (Liu et al., 2014) and one original public high-throughput sequencing data GSE98622 (Liu et al., 2017) downloaded from Gene Expression Omnibus (GEO) database^1^. Five significant modules and 21 hub genes, such as Havcr1, Krt20, Sox9, Egr1, Timp1, Serpine1, Edn1, and Apln, were screened. By experiment validation, several crucial genes were identified in AKI, such as Havcr1, Krt20, Sox9, Egr1, Timp1, Serpine1, Edn1, and Apln. These genes were very important in the process of AKI, which could be further utilized to explore novel diagnostic and therapeutic strategies.

## Materials and Methods

### High-Throughput Data and Normalization

In order to identify the key biomarkers in AKI, we used the keywords “AKI, mouse” to search on the GEO DataSets database^1^ and downloaded the gene expression profiles of GSE52004 (Liu et al., 2014) and GSE98622 (Liu et al., 2017). Both two series contained samples of mouse kidney IRI model of AKI and sham (control) group. We extracted three group sets in GSE52004 and one group set in GSE98622; each group set contained two groups, AKI 24 h group and the sham 24 h group. The detailed information of four group sets extracted from these two series is in Table 1. GSE52004 was based on the Affymetrix GPL6246 platform [(MoGene-1_0-st) Affymetrix Mouse Gene 1.0 ST Array (transcript gene version)] and provided the raw data. The preprocessing of the gene expression profile data, which includes the background correction, the quantile normalization, the median polish summarization, and the log2 transformation, was performed by R software^2^ and RStudio software^3^ using the Robust Multichip Average (RMA) algorithmin affy package, which can be downloaded on the Bioconductor website^4^ (Lin and Lin, 2017). GSE98622 has already provided the RMA-normalized data. The preprocessed data were filtered to include only those probe sets with annotations and export as expression data. Probe set annotation files (^∗^.transcript.csv) were download on the official website of Affymetrix^5^. These CSV files contain the design–time data of the high throughput, such as gene symbol and genomic location. We then performed principal component analysis (PCA) by scatterplot3d package and princomp function in R software.

**TABLE 1:** Details for GroupSet extracted from two AKI series.

**Name of GroupSet**	**AKI 24 h group samples**	**Sham 24 h group samples**	**GEO series**	**Characters of samples**
GroupSet A	GSM1257101, GSM1257102, GSM1257103	GSM1257107, GSM1257108, GSM1257109	GSE52004	Tubular specific CRE-driver mice
GroupSet B	GSM1257110, GSM1257111, GSM1257112, GSM1257113	GSM1257114, GSM1257115	GSE52004	CRE recombinase-dependent activation of an eGFP-tagged L10a ribosomal protein subunit mice
GroupSet C	GSM1257116, GSM1257117, GSM1257118, GSM1257119	GSM1257120, GSM1257121	GSE52004	C57BL/6 mice
GroupSet D	GSM2602045, GSM2602046, GSM2602047	GSM2602033, GSM2602034, GSM2602035	GSE98622	C57BL/6 mice

*AKI, Acute Kidney Disease; GEO, Gene Expression Omnibus.*

### Identification of Differentially Expressed Genes

The linear model for high-throughput data analysis (limma) in Bioconductor was applied to find DEGs by comparing expression value between AKI 24 h samples and sham 24 h samples in the four group sets. Differential expression was calculated using an empirical Bayes model. The criteria for the statistically significant difference of DEGs was | log2 fold change (FC)| ≥ 1 in expression and adjusted *p-*value (false discovery rate, FDR) < 0.05. Volcano plot of all DEGs was performed by ggplot2 package in R. The overlapping DEGs among four group sets were further analyzed by VennDiagram package in R software.

### GO and KEGG Pathway Enrichment Analyses of DEGs

The Database for Annotation, Visualization and Integrated Discovery (DAVID)^6^ is a gene functional classification tool that integrates a set of functional annotation tools for investigators to analyze biological functions behind massive genes (Huang et al., 2009). GO and KEGG pathway enrichment analyses of 722 DEGs were performed based on DAVID online tool. In the GO enrichment analysis, the categories include the cellular component (CC), the biological process (BP), and the molecular function (MF) terms (Zhang et al., 2017), and adjusted *p* < 0.05 was regarded as statistically significant differences. In the KEGG pathway enrichment analysis, enriched pathways were identified according to the hyper geometric distribution with an adjusted *p* < 0.05.

### PPI Network Construction and Analysis of Modules

Considering that proteins rarely work alone, it is necessary to study the interactions among proteins. The Search Tool for the Retrieval of Interacting Genes/Proteins (STRING)^7^ is an online biological resource database that is commonly used to identify the interactions between known and predicted proteins (Szklarczyk et al., 2015). By searching the STRING database, the PPI network of the 722 overlapping DEGs were selected with a score > 0.7, and the PPI network was visualized by Cytoscape software (Shannon et al., 2003)^8^. In the PPI network, each node stands for a gene or a protein, and edges represent the interactions between the nodes. We then used the plug-in named Molecular Complex Detection (MCODE) and ClusterOne (Nepusz et al., 2012) by Cytoscape to filter the modules of the PPI network. The number of nodes > 6 and MCODE scores > 5 were set as the criteria (Lin and Lin, 2017).

### Identification of Hub Genes

Hub genes/proteins are a few numbers of the proteins/DEGs (hubs) that have more association with other DEGs/proteins. They are the important nodes in the PPI network. To find the hub genes of the PPI network of overlapping DEGs, Cyto-Hubba (Chin et al., 2014) and cytoNCA (Tang et al., 2015), both java plugin for Cytoscape software, were applied. In this study, we mined the top 21 hub genes by combining the above two methods, The PPI network of these 21 hub genes was constructed and visualized based on GeneMANIA (Franz et al., 2018) plugin in Cytoscape. GO and KEGG pathway enrichment analyses were performed for hub genes. An adjusted *p* < 0.05 was considered statistically significant.

### Animals and Procedures

C57BL/6 mice (20–25 g) were purchased from the Animal Center of Chinese PLA General Hospital. All animal procedures were approved by the Institutional Animal Care and Use Committee at the Chinese PLA General Hospital and Military Medical College. The 12 male mice were randomly assigned to two groups: 6 mice underwent bilateral renal ischemia and reperfusion surgery (the AKI group), and the remaining 6 mice underwent sham surgery (the sham group). Renal ischemia (45 min) and reperfusion and renal sham surgery were performed as described previously (Hu et al., 2013). At 24 h after reperfusion, blood and kidney samples were harvested for further processing.

### Assessment of Kidney Injury

Kidney injury was assessed by measuring the levels of serum creatinine and blood urea nitrogen and calculating the changes in levels. Blood samples were collected from the vena cava at the indicated time points, and the serum was separated by centrifugation at 3,000 rpm for 15 min at 4°C and then sent to the PLA General Hospital Biochemistry Department to detect serum Cr and BUN.

### Histopathological Examination

A quarter of the kidney was fixed in 4% formaldehyde, dehydrated, and embedded in paraffin. Tissue sections (4 mm) were stained with periodic acid–Schiff (PAS). Histological examinations were performed in a blinded manner for acute tubular necrosis (ATN) scores regarding the grading of tubular necrosis, cast formation, tubular dilation, and loss of brush border as described before (Hu et al., 2013). Fifteen non-overlapping fields (400×) were randomly selected and scored as follows: 0, none; 1, 1–10%; 2, 11–25%; 3, 26–45%; 4, 46–75%; and 5, > 76%.

### Quantitative Real-Time PCR

Frozen tissue samples were lysed in TRIzol reagent (Invitrogen, Carlsbad, CA, United States), and total RNA was extracted according to the manufacturer’s instructions. The levels of transcripts were determined by quantitative real-time PCR (qRT-PCR) using TransStart^TM^ Top Green qPCR SuperMix (AQ131, Transgen, Beijing, China) on an Applied Biosystems 7500 system PCR cycler (Applied Biosystems, Foster City, CA, United States). The data were normalized to 18S expression and further normalized to the control group. Primers were obtained from Genomics (BGI Tech, China). All of these primers are listed in Table 2.

**TABLE 2:** Sequences of oligonucleotide primers used for quantitative real-time PCR (RT-PCR).

**Gene name**	**Forward primer**	**Reverse primer**
Agt	GGCGCTGAAGGATACACAG	GGCTCGAACGTTGACTCTGG
Anxa1	AGGAGCTTTCCTCATCTTCGC	CACCCTTCATGGCTTCGTACA
Atf3	AGGCAGGAGCATCCTTTGTC	CTGCTTTGCATAGGACCCCA
Casr	ACAGTTGCCTTGTGATCCTC	TGTAGAGACTGCCCGAGATGT
Clu	GGTGCATTCTCCGGCATTC	GGAGAATCTTCATGGCGTGG
Crct1	GGGTGGGACTTCAGATCACG	GCGACAATCAAATCAGGAGGC
Crisp1	TTGTCCTGTTGGCAATTATCAAGG	ACAACTGGCACACGGTTCTC
Ctgf	AGACCTGTGCCTGCCATTAC	ACGCCATGTCTCCGTACATC
Edn1	AGAAGTTGACGCACAACCGA	GGGAACACCTCAGCCTTTCT
Egr1	AGTTTCACGTCTTGGTGCCT	AAGGCTAAGGTGAGCGTGTC
F2r	GCGTGGTCATTTGGGTGATG	TGGCAGGTGGTGATGTTGAG
Fga	GTCACAGGTCCTGATGGTCG	CCCAGAAAGGTCAGGATGCC
Fgb	CAAGGCTACTGCCAACCAGA	CCGTAGGACACAACACTCCC
Fgg	CTCCATCGGAGAAGGACAGC	TCCTGAAAGTCCATTGTCCCA
Fos	GGGAATGGTGAAGACCGTGT	CCGTTCCCTTCGGATTCTCC
Fosl1	GCAAGTGGTTCAGCCCAAGA	CTGGGCTGGATGTTCGGTAG
Grp	CAACGCACTCTCAGCCTAGT	GCGCGGATACATCTTAGCCA
Gsta1	AAGCAAGGAAGGCTTTCAAGATTCA	AAACCATTAGAGGCCAGTATCTGTG
Il24	TGCACAAGAAGAACCAGCCA	TGGCAAGACCCAAATCGGAA
Havcr1	ACATATCGTGGAATCACAACGAC	ACAAGCAGAAGATGGGCATTG
Krt20	GCAGTGGTACGAAACCAACG	CTGCAGCCAGCTTAGCATTG
Lcn2	TTTGTTCCAAGCTCCAGGGC	ACTGGTTGTAGTCCGTGGTG
Mmp3	ATGAAGGGTCTTCCGGTCCT	CTGTCATCTCCAACCCGAGG
Myc	CCCCAAGGGAAGACGATGAC	TGAAGGTCTCGTCGTCAGGA
Pdgfb	CCAAAGGCAAGCACCGAAAG	CGTCCGAATCAGGCATCGAG
Pros1	TCCTCTCAGCAATGAGGGTC	AGGACTTGTGAAGCACGCTC
Ptgs2	AGCCCATTGAACCTGGACTG	ACCCAATCAGCGTTTCTCGT
Qsox1	CACTGCCCTAGATGTACCAGC	AGGCTTCAGTGTCTCTCTTGC
Serpine1	TCTCTTTGTGGTTCGGCACA	TTCGTCCCAAATGAAGGCGT
Sox9	GTGCAAGCTGGCAAAGTTGA	TGCTCAGTTCACCGATGTCC
Sprr2f	GGAATACTTTGGAGAACCTGATCC	TTTGGTGGTGGACACACAGGA
Sult1e1	TTCCACGGGAACATCTGGAC	GAACTCCACGGAACTCTCCA
Tgfb1	AGGGCTACCATGCCAACTTC	CCACGTAGTAGACGATGGGC
Timp1	CCCCAGAAATCAACGAGACCA	ACTCTTCACTGCGGTTCTGG
Vgf	GTGACACCGGCTGTCTCTG	AAGCAGAAGAGGACGGATGC

### Western Blot Analysis

EGR1 antibody (MA5-15008, Invitrogen, Thermo Fisher Scientific, United States), glyceraldehyde 3-phosphate dehydrogenase (GAPDH) mouse monoclonal antibody (AF0006, Beyotime Biotechnology, China), horseradish peroxidase (HRP)-labeled goat antirabbit immunoglobulin G (IgG) (H + L) antibody (A0208, Beyotime Biotechnology, China), and HRP-labeled goat antimouse IgG (H + L) antibody (A0216, Beyotime Biotechnology, China) were used for Western blot analysis. GAPDH served as control. Approximately 30 μg of protein of each mouse was added to validate.

### Immunofluorescence Analysis

Kidneys were fixed for 18 h in 4% paraformaldehyde (PFA) at 4°C, then incubated 2 h in 30% sucrose in phosphate-buffered saline (PBS) and embedded in OCT. The embedded kidney was cut into 5 μm thick sections and washed with PBS thrice for 5 min, and then permeabilized with 1% Triton X-100 buffer. After washed with PBS thrice for 5 min, the sections were blocked with 10% casein (SP5020, Vector, Burlingame, CA, United States) and incubated with primary antibodies specific for EGR1 (MA5-15008, Invitrogen, Thermo Fisher Scientific, United States) overnight in a moisture chamber and then washed with PBS to remove unbound primary antibody. Next, the sections were incubated with Cy3-labeled goat antirabbit IgG (H + L) antibody (A0516, Beyotime Biotechnology, China) and fluorescein-labeled Lotus Tetragonolobus Lectin (LTL, FL1321, Vector Laboratories, United States) for 60 min at room temperature and washed as described for the primary antibody. Finally, the sections were washed with PBS and added with 4′,6-diamidino-2-phenylindole (DAPI) (ab104139, Abcam, Cambridge, MA, United States).

## Results

### Preprocessing of High-Throughput Data

**FIGURE 1:**
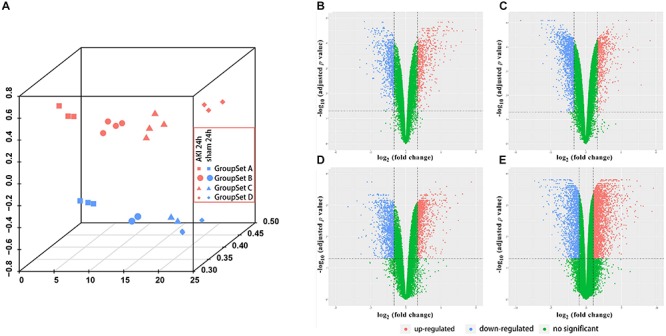
Principle component analysis (PCA) plot and differential expression data between AKI 24 h and sham 24 h groups in all four GroupSets. **(A)** PCA plot shows the tight clustering of biological replicates and distinct clustering between AKI 24 h and sham 24 h conditions in all four GroupSets. Differential expression data between AKI 24 h and sham 24 h groups in **(B)** GroupSet A data, **(C)** GroupSet B data, **(D)** GroupSet C data, and **(E)** GroupSet D data. The criteria for statistically significant difference of DEGs was adjusted *p* < 0.05 and |log2 fold change (FC)| ≥ 1 in expression.

Before the next analysis, we performed the quality control for every raw file (group sets A, B, and C) to guarantee the quality of every chip. The box plot results before and after normalization are shown in Supplementary Figure S1. We did not perform the box plot for group set D since GSE98622 has already provided the RMA-normalized data. The preprocessed data were filtered to include only those probe sets with annotations and export as expression data. Next, we applied PCA to the AKI 24 h group and sham-surgery 24 h group for all four group sets (Figure 1A). All biological replicates for eight groups (different shapes and colors) showed good clustering, indicative of a high degree of similarity in each group. We can also find tight clustering of biological replicates and distinct clustering between AKI 24 h and sham 24 h conditions (different colors) in all four group sets.

### Identification of DEGs

**FIGURE 2:**
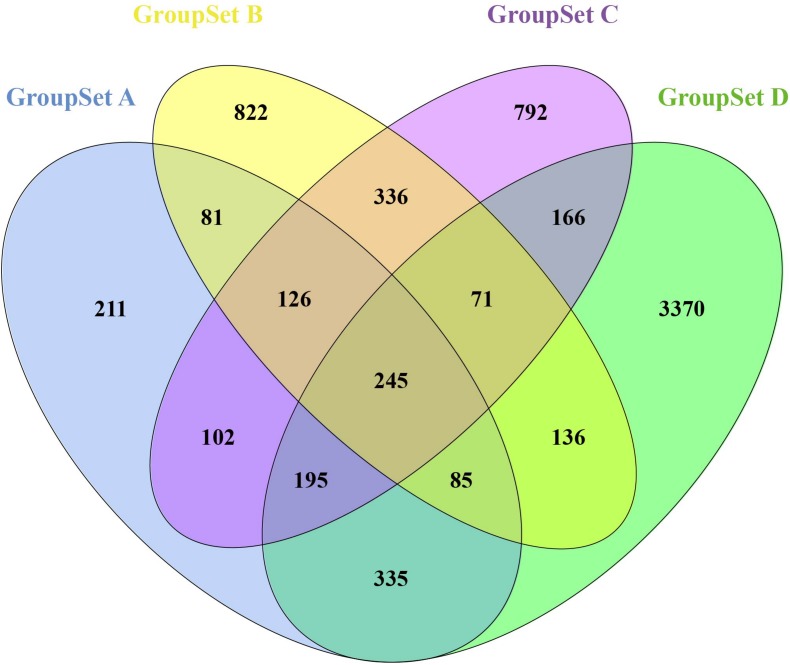
Identification of overlapping DEGs in all four GroupSets. Each colored ellipse in the Venn diagram represents a GroupSet, and the number stands for the number of DEGs in each cross area.

A total of 1,380 DEGs were obtained from the group set A (Supplementary Table S1A); among them, 646 genes were upregulated, and 734 genes were downregulated. Overall, 1,902 DEGs were screened from the group set B (Supplementary Table S1B); among them, 605 genes were upregulated, and 1,297 genes were downregulated. In addition, 2,033 DEGs were identified from the group set C (Supplementary Table S1C), including 951 upregulated genes and 1,082 downregulated genes. What is more, 4,603 DEGs were screened from the group set D (Supplementary Table S1D), including 2,973 upregulated genes and 1,630 downregulated genes. The differential expression of multiple genes in each of the four group sets is shown in Figures 1B–E. The cluster heatmap results of the top 50 upregulated and 50 downregulated DEGs are shown in Supplementary Figure S2. We then performed overlapping DEG analysis among the top 50 upregulated DEGs of four group sets; the result indicated that Krt20, Serpine1, Sprr2f, and Timp1 showed identical expression trends in all four group sets, and Fosl1, Gsta1, Havcr1, Plaur, Runx1, Slc7a11, Sox9, and Sprr2g were differentially expressed in at least three group sets. We also performed overlapping DEGs analysis among all the DEGs of these four group sets by VennDiagram package (Figure 2); among them, 245 DEGs showed identical expression trends in all four group sets, including 157 upregulated genes and 88 downregulated genes (Supplementary Table S2), and 722 DEGs were differentially expressed in at least three group sets.

### GO and KEGG Pathway Enrichment Analyses of 722 DEGs

**FIGURE 3:**
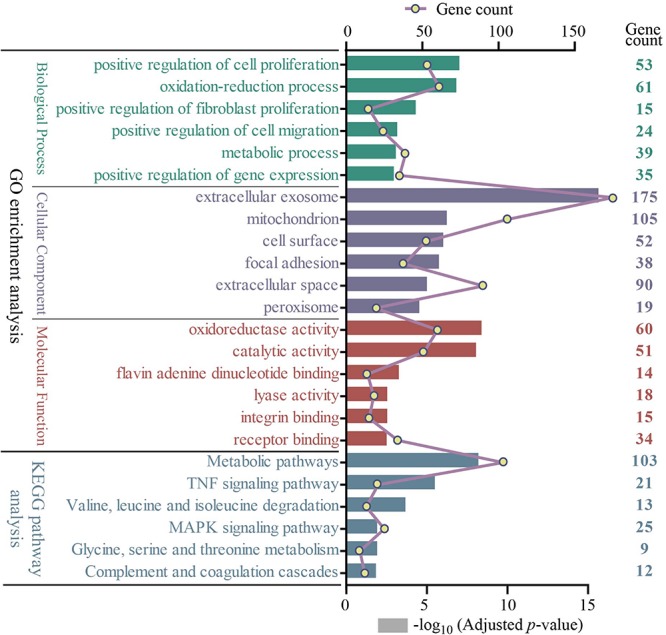
The GO and KEGG pathway enrichment analysis of 722 overlapping DEGs (top 6 were listed). Four different colors separately represent four different categories: biological process, cellular component, molecular function, and KEGG pathway. The rectangle represents−log10 (adjusted *p-*value); the line and point represent gene count. GO, Gene Ontology; KEGG, Kyoto Encyclopedia of Genes and Genomes.

All 722 overlapping DEGs that were differentially expressed in at least three group sets were uploaded into the online software DAVID to evaluate the GO and KEGG pathways. GO analysis of DEGs was divided into three functional groups, including biological processes, cellular component composition, and molecular function. Significant results of the GO and KEGG pathway enrichment analyses of DEGs are shown in Figure 3. In the biological process group, 722 DEGs were mainly enriched in the positive regulation of cell proliferation, oxidation–reduction process, positive regulation of fibroblast proliferation, positive regulation of cell migration, metabolic process, and positive regulation of gene expression. In the cellular component group, 722 DEGs were mainly enriched in the extracellular exosome, mitochondrion, cell surface, focal adhesion, extracellular space, and peroxisome. In the molecular function group, 722 DEGs were mainly enriched in the oxidoreductase activity, catalytic activity, flavin adenine dinucleotide binding, lyase activity, integrin binding, and receptor binding. The KEGG pathway results revealed that the DEGs are significantly enriched in metabolic pathways; tumor necrosis factor (TNF) signaling pathway; valine, leucine, and isoleucine degradation; MAPK signaling pathway; glycine, serine, and threonine metabolism; and complement and coagulation cascades (Supplementary Table S3).

### PPI Network Construction and Analysis of Modules

**FIGURE 4:**
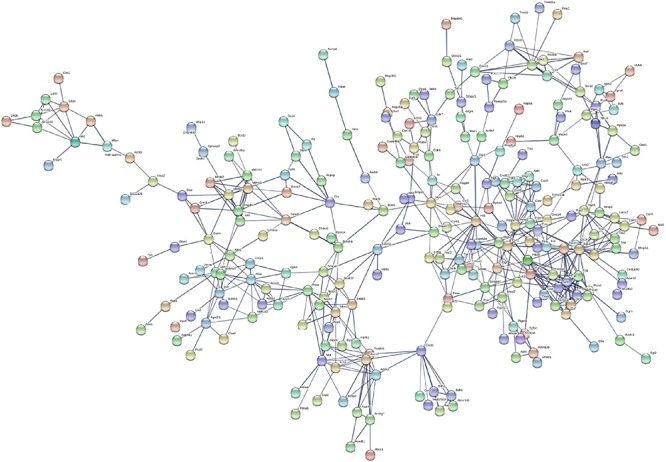
Protein–protein interaction network of the 722 overlapping DEGs with a score >0.7. Disconnected nodes were hiding in the network. Each node stands for a gene or a protein, and edges represent the interactions between the nodes.

**FIGURE 5:**
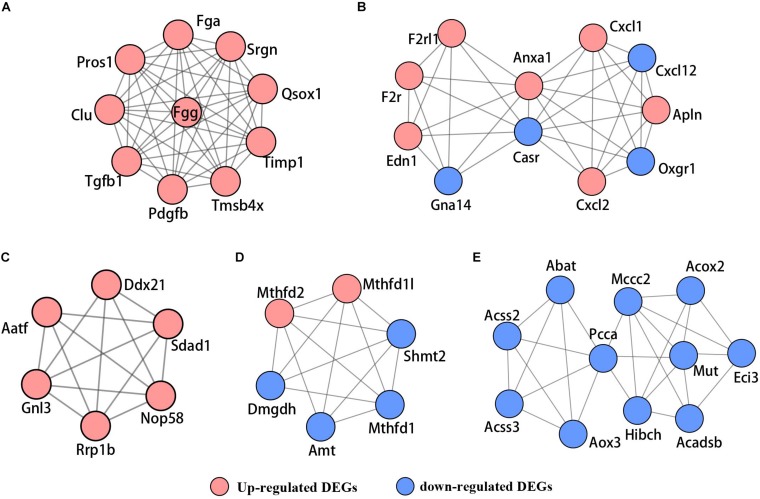
The PPI network of five significant modules selected by MCODE. **(A)** Module 1, **(B)** module 2, **(C)** module 3, **(D**) module 4, and **(E)** module 5. Red nodes represent upregulated genes; blue nodes stand for downregulated genes.

Based on the STRING database, the PPI network of the 722 overlapping DEGs was selected; 312 nodes and 585 edges were established in the PPI network with score > 0.7 (Figure 4). After removing the disconnected nodes in Cytoscape software, a total of 251 nodes and 527 edges were analyzed. Five modules met the criteria of MCODE score > 5 and the number of nodes > 6; the results are shown in Figure 5. All of these modules got a *p* < 0.01, and module 1 was still the most significant in ClusterOne plug-in. KEGG pathway of the five modules are listed in Supplementary Table S4. The results showed that the DEGs included in these five modules were mainly associated with metabolic pathways (including 6 genes from module 4 and 10 genes from module 5).

### Identification of Hub Genes

**FIGURE 6:**
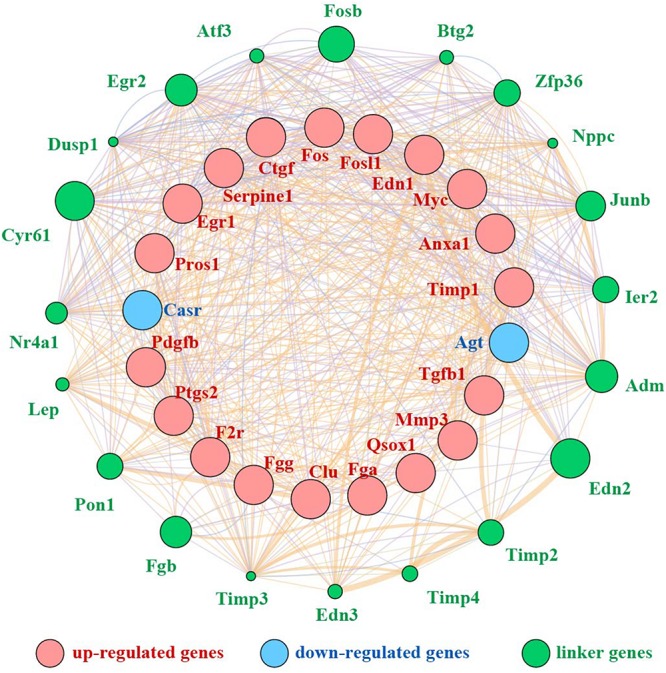
PPI network of 21 hub genes. Red nodes represent upregulated genes, blue nodes stand for downregulated genes, and green nodes represent the linker genes.

We mined the top 21 hub proteins by combining Cyto-Hubba and cytoNCA. These hub genes included tissue inhibitor of metalloproteinase 1 (Timp1), Quiescin Q6 sulfhydryl oxidase 1 (Qsox1), transforming growth factor beta 1 (Tgfb1), platelet-derived growth factor, B polypeptide (Pdgfb), protein S alpha (Pros1), matrix metallopeptidase 3 (Mmp3), fibrinogen alpha chain (Fga), fibrinogen gamma chain (Fgg), clusterin (Clu), serine peptidase inhibitor (Serpine1), coagulation factor II receptor (F2r), connective tissue growth factor (Ctgf), endothelin1 (Edn1), calcium-sensing receptor (Casr), annexinA1 (Anxa1), angiotensinogen (Agt), prostaglandin-endoperoxide synthase 2 (Ptgs2), myelocytomatosis oncogene (Myc), Fos-like antigen 1 (Fosl1), FBJ osteosarcoma oncogene (Fos), and Early growth response 1 (Egr1). All the hub genes were analyzed for the GO and KEGG pathway enrichment analyses (Table 3). The PPI network of these 21 hub genes was constructed and visualized based on the GeneMANIA plugin in Cytoscape (Figure 6). The network revealed that there were strong interactions among these hub genes, and their interactions may have an effect on the AKI pathophysiological process.

**TABLE 3:** GO and KEGG enrichment analysis of 21 hub genes.

**Category**	**Pathway ID**	**Pathway term**	**Ajusted *p*-value**	**Gene count**	**Genes**
KEGG	mmu04610	Complement and coagulation cascades	0.001254913	5	Fgg, Fga, Serpine1, Pros1, F2r
	mmu05166	HTLV-I infection	0.007973337	6	Egr1, Fos, Pdgfb, Myc, Fosl1, Tgfb1
	mmu05200	Pathways in cancer	0.027346417	6	Fos, Ptgs2, Pdgfb, Myc, Tgfb1, F2r
	mmu04668	TNF signaling pathway	0.028768196	4	Fos, Ptgs2, Edn1, Mmp3
BP	GO:0008284	Positive regulation of cell proliferation	5.84E−08	11	Casr, Ptgs2, Pdgfb, Ctgf, Agt, Clu, Edn1, Myc, Tgfb1, F2r, Timp1
	GO:0045907	Positive regulation of vasoconstriction	4.39E−07	6	Fgg, Casr, Ptgs2, Fga, Pdgfb, F2r
	GO:0048146	Positive regulation of fibroblast proliferation	1.81E−04	5	Pdgfb, Agt, Serpine1, Myc, Tgfb1
	GO:0070374	Positive regulation of ERK1 and ERK2 cascade	3.02E−04	6	Fgg, Fga, Pdgfb, Ctgf, Tgfb1, F2r
	GO:0048661	Positive regulation of smooth muscle cell proliferation	2.50E−04	5	Egr1, Ptgs2, Pdgfb, Edn1, Myc
	GO:0007596	Blood coagulation	2.54E−04	5	Fgg, Fga, Pdgfb, Pros1, F2r
CC	GO:0005615	Extracellular space	1.14E−08	14	Fgg, Fga, Pdgfb, Ctgf, Agt, Edn1, Clu, Serpine1, Anxa1, Mmp3, Qsox1, Pros1, Tgfb1, Timp1
	GO:0005576	Extracellular region	6.52E−07	13	Fgg, Fga, Pdgfb, Ctgf, Clu, Edn1, Serpine1, Anxa1, Mmp3, Qsox1, Pros1, Tgfb1, Timp1
	GO:0072562	Blood microparticle	5.25E−06	6	Fgg, Fga, Agt, Clu, Pros1, Tgfb1
	GO:0009986	Cell surface	3.75E−05	8	Fgg, Casr, Fga, Pdgfb, Clu, Anxa1, Tgfb1, F2r
	GO:0031012	Extracellular matrix	1.57E−04	6	Pdgfb, Clu, Serpine1, Mmp3, Tgfb1, Timp1
	GO:0043234	Protein complex	0.004754681	6	Ptgs2, Clu, Anxa1, Mmp3, Myc, Pros1
MF	GO:0005102	Receptor binding	0.008833709	6	Fgg, Fga, Pdgfb, Edn1, Serpine1, F2r
	GO:0008083	Growth factor activity	0.030454845	4	Pdgfb, Ctgf, Tgfb1, Timp1
	GO:0030674	protein binding, bridging	0.075352205	3	Fgg, Fga, Anxa1
	GO:0000979	RNA polymerase II core promoter sequence-specific DNA binding	0.068271512	3	Egr1, Fos, Myc

*KEGG Kyoto Encyclopedia of Genes and Genomes, BP biological process, CC cellular component, MF molecular function. 21 hub genes: Timp1, Pdgfb, Edn1, Anxa1, Casr, F2r, Fos, Tgfb1, Pros1, Fgg, Fga, Clu, Egr1, Qsox1, Agt, Serpine1, Ptgs2, Myc, Ctgf, Mmp3, Fosl1.*

### Validation of 21 Hub Genes and 14 Other Important Upregulated Genes

**FIGURE 7:**
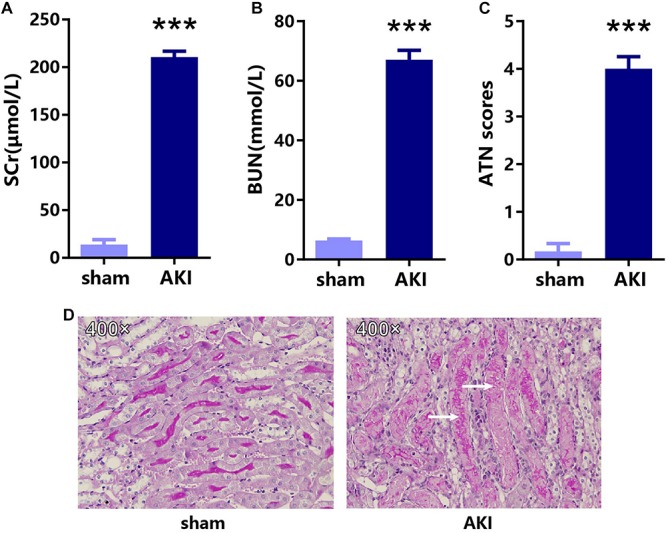
The results of SCr, BUN, and ATN scores, and PAS staining in 24 h after IRI or sham surgery. The results of **(A)** SCr, **(B)** BUN, **(C)** ATN scores, and **(D)** PAS staining in AKI and sham groups. ***p < 0.001. SCr, serum creatinine; BUN, blood urea nitrogen; ATN, tubular necrosis; PAS, Periodic acid–Schiff.

**FIGURE 8:**
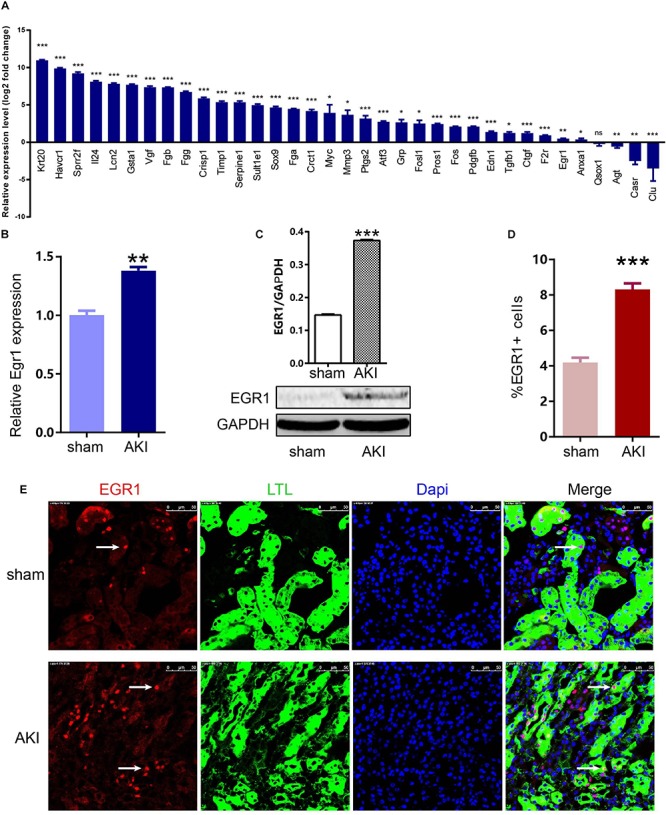
Expression of 35 key genes and EGR1 in AKI samples and sham samples. **(A)** Transcriptional levels of 35 important genes including 21 hub genes and 14 other important upregulated genes in our own AKI samples. **(B)** Translational levels of Egr1 in AKI samples and sham samples. **(C)** Western blot of EGR1. **(D,E)** Immunofluorescence of EGR1. *p < 0.05, **p < 0.01, ***p < 0.001.

To confirm the key genes identified above, we performed IRI surgery in mice. The results of SCr, BUN, and ATN scores, and PAS staining in 24 h after IRI or sham surgery are shown in Figure 7. The qRT-PCR analysis was performed to validate the expression of 35 key genes including 21 hub genes and 14 other important upregulated genes in our own AKI samples (Figure 8A). Through validation, 31 genes were validated as significantly upregulated, 3 genes were significantly downregulated, and 1 gene (Qsox1) was found not significant in our own AKI samples. Krt20 was identified as the top 1 upregulated genes with a log2 (fold change) larger than 10 in all these 35 genes. Those results were consistent with the DEG analysis results, except Clu and Qsox1. Additionally, we validated the Egr1 gene as an upregulated gene not only in RNA level (Figure 8B) but also in protein level by Western blot (Figure 8D) and immunofluorescence analyses (Figure 8E); the result was also consistent with the above analysis. In conclusion, our results indicated that these key genes were significantly differentially expressed in AKI.

## Discussion

AKI is a global public health concern associated with high morbidity, mortality, and health-care costs (Zuk and Bonventre, 2016), and the therapeutic measures for AKI are still limited (Bellomo et al., 2012). Therefore, it is very necessary to explore the molecular mechanisms and develop available treatment strategies for AKI. High throughput and high-throughput sequencing technologies have been widely used to explore potential targets for AKI diagnosis and treatment. At present, many basic research papers on the mechanisms underlying the occurrence of AKI have been reported (Kumar et al., 2015; Brossa et al., 2018), but the treatment remains very limited; one of the important reason is that the results of most studies were generated from a single-cohort study or focused on merely a single event.

In this study, we integrated four group sets that contain the AKI 24 h group and sham 24 h group data, and used R software and bioinformatics to deeply analyze these datasets. At first, we

preprocessed the raw data; the box plot results showed that these data have a good quality after preprocessing (Supplementary Figure S1). PCA results showed that all of the biological replicates for the eight groups showed well clustering, which indicates a high degree of similarity in each group (Figure 1A). Volcano plots (Figures 1B–E) showed the differentially expressed genes between AKI 24 h and sham 24 h groups in four group sets. The cluster heatmaps (Supplementary Figure S2) showed the top 50 upregulated and 50 downregulated DEGs in four group sets, which indicated that the AKI and sham groups could be distinguished by the upregulated and downregulated genes.

We then performed overlapping DEGs analysis among the top 50 upregulated DEGs of each four group sets; the result indicated that Krt20, Serpine1, Sprr2f, and Timp1 showed identical expression trends in all four group sets; Fosl1, Gsta1, Havcr1, Plaur, Runx1, Slc7a11, Sox9, and Sprr2g were differentially expressed in at least three group sets. These commonly upregulated genes may play an important role in the pathology and progress of AKI.

Furthermore, we performed integrated analysis among all the DEGs of these four group sets by VennDiagram package (Figure 2); a set of 722 overlapping DEGs that were differentially expressed in at least three group sets were identified. We then performed GO and KEGG pathway enrichment analyses of these 722 overlapping DEGs. As shown in Figure 3, many genes involved in the cell proliferation, fibroblast proliferation, positive regulation of gene expression, oxidoreductase activity, metabolic pathways, and TNF signaling pathway were activated after AKI; these results were confirmed with the pathology of AKI (Bellomo et al., 2012).

In addition, we performed a PPI network of these 722 overlapping DEGs with a score > 0.7 (Figure 4) and then mined five significant modules (Figure 5) and the top 21 hub proteins in the PPI network (Figure 6). The top 2 modules were module 1, which included 10 genes, and module 2, which included 11 genes. Nine of the top 21 hub genes, Timp1, Fgg, Serpine1, Edn1, Ctgf, Ptgs2, Myc, Fosl1, and Egr1, appeared once or more in the top 50 upregulated DEGs of the four group sets. Twelve of the top 21 hub genes, Timp1, Tgfb1, Qsox1, Pros1, Pdgfb, Fgg, Fga, Clu, F2r, Edn1, and Casr, Anxa1, also appeared in the top 2 modules, indicating the importance of these genes. In the GO and KEGG pathway enrichment analyses of the top 21 hub genes, 11 genes were enriched in positive regulation of cell proliferation.

To confirm the key genes identified above, we performed IRI surgery in mice. The qRT-PCR analysis of 35 key genes including 21 hub genes and 14 other important upregulated genes in our own AKI samples (Figure 8A) showed that 31 genes were validated as significantly upregulated, 3 genes were significantly downregulated, and Krt20 were identified as the top 1 upregulated genes with a log2 (fold change) larger than 10 in all these 35 genes. Gehrke et al. (2019) reported that Egr1 was a pioneering gene that can directly regulate other early wound-induced genes. Then, we focused on Egr1, to validate its expression not only in RNA level (Figure 8B) but also in protein level by Western Blot (Figure 8D) and immunofluorescence analyses (Figure 8E). These results showed that Egr1 was significantly upregulated in AKI 24 h kidney samples. In conclusion, our results indicated that these key genes were significantly differentially expressed in AKI.

As validated by our own AKI samples, these 21 hub genes and 14 other important upregulated genes, such as Havcr1, Sox9, Egr1, Timp1, Serpine1, Edn1, and Apln, are very important in the process of AKI, which could be used to explore some new diagnostic and therapeutic strategies. For example, Havcr1, which is also known as kidney injury molecule 1 (Kim-1), is a type 1 membrane protein. It was widely known as a biomarker for tubular injury and repair processes (Ichimura et al., 2004; Lim et al., 2013). Sex-determining region Y-box 9 (Sox9) played an important role in the development of many organ systems and cell types (Reginensi et al., 2011). Kumar et al. (2015) identified that Sox9 activation acts as a quick response to AKI within recovering cells of the damaged segment of the proximal tubule, and also Sox9 was required for the physiological repairing process. Kang et al. (2016) identified Sox9 as a cell marker of segment-specific progenitor in the recovering kidney, and cells that express Sox9 proliferated and became the loop of Henle, distal tubule, and proximal tubule after AKI. Ma et al. (2018) reported that Sox9 + cells had a proliferative capacity and could regenerate epithelial cells in the proximal tubule in a mouse unilateral partial nephrectomy model, and Sox9 activation was involved with Notch signaling pathway. Therefore, Sox9 plays a pivotal role in AKI. Timp1, which is a representative member of the TIMP family, is expressed in several tissues that act as an inhibitor of matrix metalloproteinases. The main biological function of TIMPs is to inhibit MMPs by forming a 1:1 enzyme-inhibitor complex (Guo et al., 2016). Bojic et al. (2015) reported that Timp1 was a good diagnostic biomarker of sepsis-associated AKI and may involve in the pathogenesis of sepsis and septic shock. Uehara et al. (2013) successfully identified Timp1 as a candidate genomic biomarker for the early detection of acute papillary injuries in rats.

Serpine1, also named as plasminogen activator inhibitor type-1 (PAI-1), is a very strong proteolytic enzyme. Serpine1 is regulated by various signals, including many growth factors, glucose, hormones, cytokines, catecholamine, and hypoxia (Kim et al., 2013). Serpine1 is produced in little amounts in healthy kidneys but is widely expressed in both AKI and CKD kidneys (Eddy and Fogo, 2006). Increased Serpine1 expression resulted in the accumulation of extracellular matrix, while inhibition of Serpine1 activity increased matrix turnover and could reduce glomerulosclerosis (Malgorzewicz et al., 2013). These results indicated that Serpine1 regulation would be a good strategy to treat ischemia–reperfusion (I/R)-induced inflammatory renal injury. Edn1, the predominant isoform of the endothelin peptide family, also known as endothelin-1 (ET-1), acts through the receptors type A (ETRA) and B (ETRB) (Zanatta et al., 2015). Studies showed that ET-1 was increased in renal tissue in AKI (Ong et al., 2015). Intervention with ETRA antagonists can slow down and prevent the progression from AKI to CKD (Zager et al., 2013) and provide dramatic protection. Kurata et al. (2004) reported that nitric oxide has protective effects against AKI through the suppression of ET-1 overproduction in the postischemic kidney. These results suggested that Edn1 was a potential therapeutic biomarker. The regulation of Edn1 would be a new strategy for the treatment of AKI. Apln, which belongs to the G-protein-coupled receptor family, also known as Apelin, is an endogenous ligand of the specific receptor APJ (Antushevich and Wojcik, 2018). Chen et al. (2015) reported that overexpression of Apelin significantly inhibited apoptosis, hypoxia-induced TGF-β1, to protect against kidney IRI. Apelin can also activate the p-ERK, p-AKT, and Sirt3, and protected against ischemic injury by activating the PI3K/AKT axis (He et al., 2015). Day et al. (2013) reported that apelin can prevent diabetic nephropathy’s progression. Pan et al. (2010) reported that apelin decreases the inflammatory response and cardiac deterioration in the sepsis model. Apelin could also prevent IRI kidney damage and decreased BUN and creatinine levels (Bircan et al., 2016). These results suggested that Apln was a potential therapeutic biomarker.

At present, a great number of studies have been performed on AKI gene expression profiles (Wei et al., 2010; Liu et al., 2014, 2017; Cui et al., 2016), but their results of DEGs are often not the same due to the heterogeneity among each independent experiment. In the present study, we perform an integrated analysis of high-throughput data from different gene expression profiles and validated the expression of 35 key genes including 21 hub genes and 14 other important upregulated genes in our own AKI samples. Those key genes may be potential molecular markers for prognosis and early detection, as well as therapeutic drug targets for treating AKI.

However, there were still some limitations in our present study. First, there is lack human kidney tissue sample data, which may result in not full applicability in human. Second, more high-throughput microarray and high-throughput sequencing need to be included to get more robust results. Third, the above results, such as the gene expression level and gene function, should be validated by further experiments.

## Conclusion

In conclusion, by integrated analysis of different high-throughput data and validation, we identified several crucial genes in AKI, such as Havcr1, Krt20, Sox9, Egr1, Timp1, Serpine1, Edn1, and Apln; they might have cofunctions, and their dysregulations might alter activities of pathways such as positive regulation of cell proliferation, complement and coagulation cascades, and TNF signaling, which might finally promote AKI progression. This study is of great value for the prediction of key regulators in the process of AKI and the identification of potential diagnostic and therapeutic biomarkers for AKI.

## Data Availability Statement

All of the original high-throughput data can be public achieved at the Gene Expression Omnibus (GEO) database (https://www.ncbi.nlm.nih.gov/geo/), and other data supporting the study is within the paper. The links to all databases and software used in this study are as follows: Affymetrix official website (http://www.affymetrix.com/support/technical/annotationfilesmain.affx), Bioconductor (http://www.bioconductor.org/), Cytoscape software (http://www.cytoscape.org/), version 3_6_1 for windows_64 bit, Database for Annotation, Visualization and Integrated Discovery (DAVID, http://david.ncifcrf.gov/), GSE52004 (https://www.ncbi.nlm.nih.gov/geo/query/acc.cgi?acc=GSE52004), GSE98622 (https://www.ncbi.nlm.nih.gov/geo/query/acc.cgi?acc=GSE98622), R software (https://www.r-project.org/), version 3.5.1 for windows_64 bit, R Studio (https://www.rstudio.com/), version 1.1.456 for windows_64bit, Affy package, version 1.50.0, Limma package, version 3.36.3, Ggolot2 package, version 3.0.0, Scatterplot3d package, version 0.3-41, VennDiagram package, version 1.6.20, Search Tool for the Retrieval of Interacting Genes//Proteins (STRING, http://string-db.org/).

## Author Contributions

XC designed the experiments. JC and YC carried out the study, collected all the important information, and completed the manuscript. AO edited the language of the manuscript. All authors read and approved the final manuscript.

## Conflict of Interest

The authors declare that the research was conducted in the absence of any commercial or financial relationships that could be construed as a potential conflict of interest.

## Footnotes

1 https://www.ncbi.nlm.nih.gov/geo/

2 https://www.r-project.org/

3 https://www.rstudio.com/

4 http://www.bioconductor.org/

5 http://www.affymetrix.com/support/technical/annotationfilesmain.affx

6 http://david.ncifcrf.gov/

7 http://string-db.org/

8 http://www.cytoscape.org/

## Supplementary Material

FIGURE S1 The box plots before and after normalization of gene expression. **(A)** The standardization of GroupSet A data, **(B)** the standardization of GroupSet B data, and **(C)** the standardization of GroupSet C data. The abscissa is the sample, and the ordinate is the Expression Value. The normalization process normalized the signal strength of all chips to an interval with similar distribution characteristics.

FIGURE S2 Hierarchical clustering map of the top 50 up-regulated DEGs and the top 50 down-regulated DEGs in four GroupSets. **(A)** GroupSet A data, **(B)** GroupSet B data, **(C)** GroupSet C data, and **(D)** GroupSet D data. The red rectangle represents up-regulated genes; the green rectangle represents down-regulated genes. Each row represents a single gene; each column represents a tissue sample. DEGs, differentially expressed genes.

TABLE S1 All of the differential expression gene information including logFC and FDR in all four GroupSets.

TABLE S2 Genes that were differentially expressed in all four GroupSets in AKI by integrated analysis of high-throughputs.

TABLE S3 GO and KEGG enrichment analysis of genes that were differentially expressed in ≥ 3 GroupSets (top 6 significantly enriched terms were listed).

TABLE S4 The KEGG pathway of five significant modules selected by MCODE.

## Abbreviations

Agt: angiotensinogen
AKI: acute kidney injury
Anxa1: annexin A1
ATN: tubular necrosis
BP: biological process
BUN: blood urea nitrogen
Casr: calcium-sensing receptor
CC: cellular component
CKD: chronic kidney disease
Clu: clusterin
Ctgf: connective tissue growth factor
DAVID, Database for Annotation: Visualization and Integrated Discovery
DEGs: differentially expressed genes
Edn1: endothelin 1
Egr1: early growth response 1
ET-1: endothelin-1
F2r: coagulation factor II receptor
FC: fold change
Fga: fibrinogen alpha chain
Fgg: fibrinogen gamma chain
Fos: FBJ osteosarcoma oncogene
Fosl1: Fos-like antigen 1
GEO: Gene Expression Omnibus
GO: Gene Ontology
IRI: ischemia–reperfusion injury
KDIGO: Kidney Disease: Improving Global Outcomes
KEGG: Kyoto Encyclopedia of Genes and Genomes
Kim-1: kidney injury molecule 1
MCODE: Molecular Complex Detection
MF: molecular function
Mmp3: matrix metallopeptidase 3
Myc: myelocytomatosis oncogene
PAI-1: plasminogen activator inhibitor type-1
PAS: periodic acid–Schiff
PCA: principal component analysis
Pdgfb: platelet-derived growth factor B polypeptide
PPI: protein–protein interaction
Pros1: protein S alpha
Ptgs2: prostaglandin-endoperoxide synthase 2
Qsox1: quiescin Q6 sulfhydryl oxidase 1
RMA: robust multichip average
SCr: serum creatinine
Serpine1: serine peptidase inhibitor
Sox9: sex determining region Y-box 9
STRING: Search Tool for the Retrieval of Interacting Genes/Proteins
Tgfb1: transforming growth factor beta 1
Timp1: tissue inhibitor of metalloproteinase 1.
