# Supplementary figures and images for: Identification and Validation of Potential Biomarkers and Their Functions in Acute Kidney Injury
Source: Front Genet. 2020 May 12;11:411. doi: 10.3389/fgene.2020.00411 (PMC7247857; doi:10.3389/fgene.2020.00411)

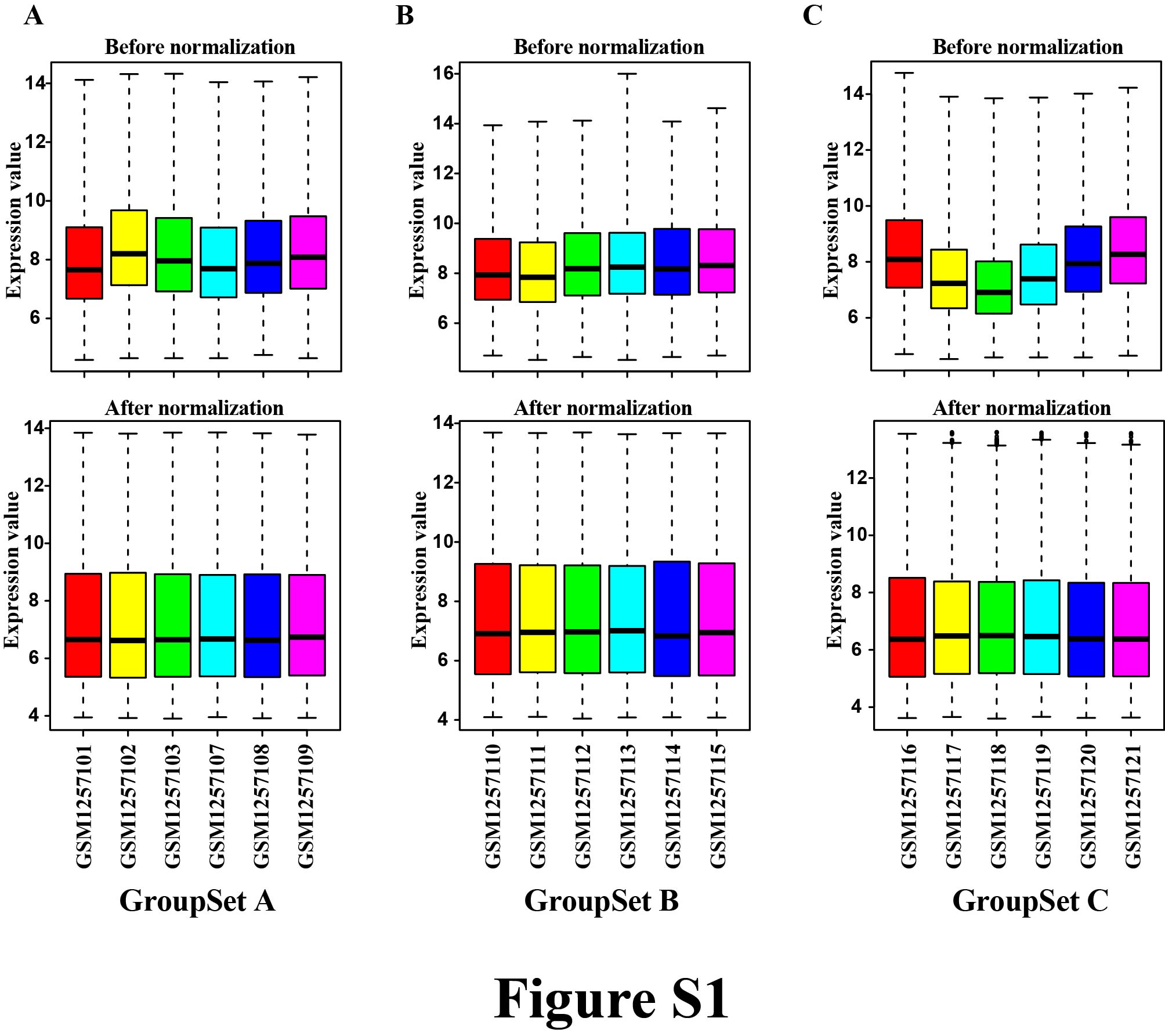

Supplement: FIGURE S1 — The box plots before and after normalization of gene expression. (A) The standardization of GroupSet A data, (B) the standardization of GroupSet B data, and (C) the standardization of GroupSet C data. The abscissa is the sample, and the ordinate is the Expression Value. The normalization process normalized the signal strength of all chips to an interval with similar distribution characteristics. [file Image_1.JPEG]

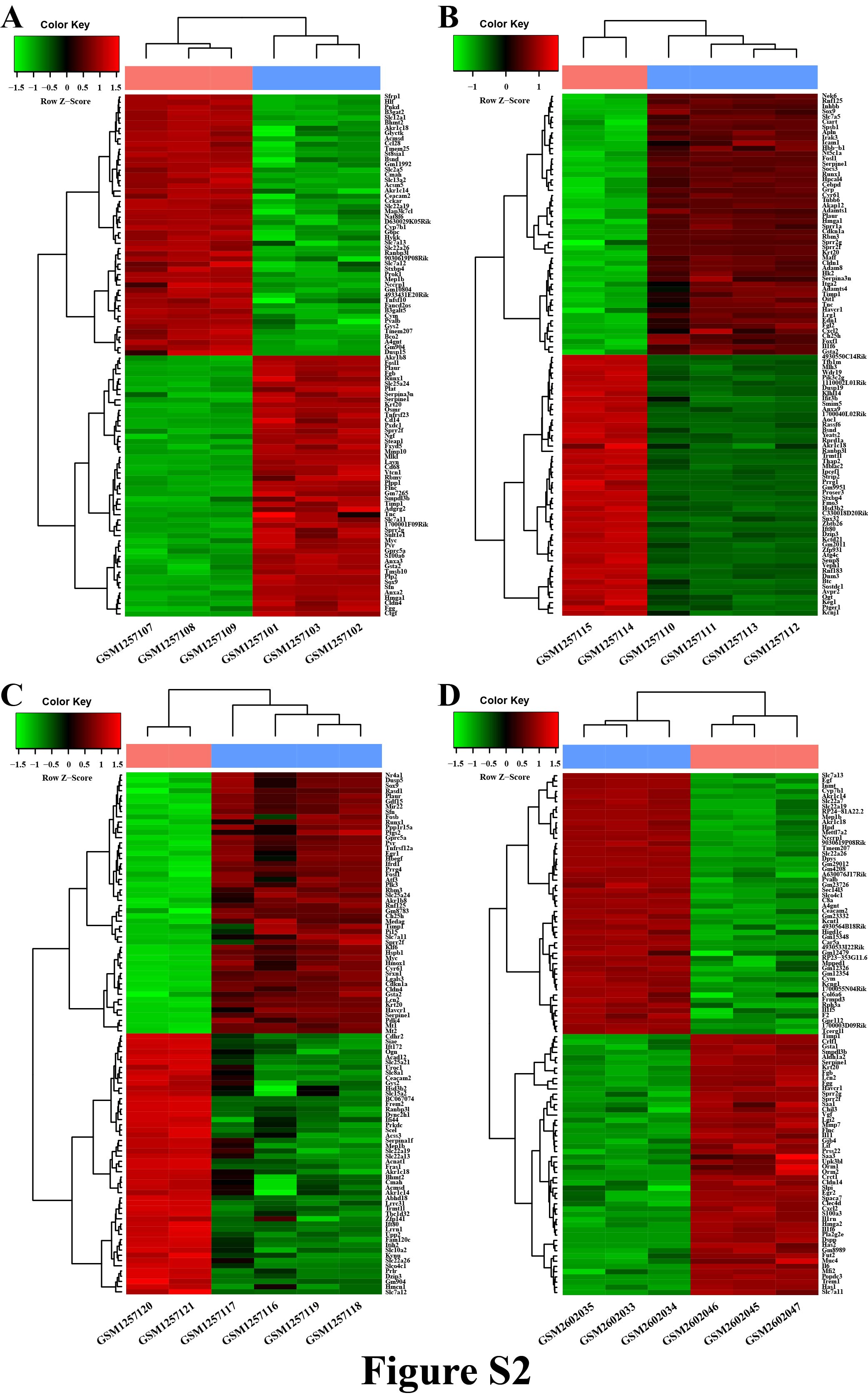

Supplement: FIGURE S2 — Hierarchical clustering map of the top 50 up-regulated DEGs and the top 50 down-regulated DEGs in four GroupSets. (A) GroupSet A data, (B) GroupSet B data, (C) GroupSet C data, and (D) GroupSet D data. The red rectangle represents up-regulated genes; the green rectangle represents down-regulated genes. Each row represents a single gene; each column represents a tissue sample. DEGs, differentially expressed genes. [file Image_2.JPEG]
