# Supplementary material for: Identification and Validation of Potential Biomarkers and Their Functions in Acute Kidney Injury
Source: Front Genet. 2020 May 12;11:411. doi: 10.3389/fgene.2020.00411 (PMC7247857; doi:10.3389/fgene.2020.00411)
Supplement: TABLE S2 — Genes that were differentially expressed in all four GroupSets in AKI by integrated analysis of high-throughputs. [file Table_2.doc]

**Table S2 Genes that were differentially expressed in all four GroupSets in AKI by integrated analysis of microarrays**

| **DEGs** | **Gene names** |
| --- | --- |
| Up-regulated  (157 genes) | Adam8, Adamts1, Adm, Akap12, Akr1b8, Aldh1a2, Aloxe3, Anxa1, Anxa3, Apobec3, Arf2, Asns, Atf3, Bcl3, Birc3, Cd14, Cd44, Cd68, Cd9, Cd93, Cebpd, Chrnb1, Clcf1, Cldn4, Cldn7, Ctgf, Ctsc, Cyr61, Ddx21, Dusp10, Dusp5, Edn1, Efhd2, Elf4, Emp1, Entpd1, Epha2, F2r, F3, Fam57a, Flnc, Fndc4, Fosl1, Fosl2, Fut2, Fxyd5, Gas7, Gdf15, Gprc5a, Havcr1, Hbegf, Hilpda, Hmox1, Hpcal4, Igf2bp2, Il1f6, Il34, Irak3, Itga5, Klf4, Klf6, Klf7, Krt19, Krt20, Lamc2, Lif, Lrp8, Lrrc32, Lrrc8c, Maff, Map3k6, Map4k4, Mapk6, Mmp3, Mthfd1l, Myc, Myh9, Nek6, Ngf, Nras, Nt5c1a, Nucb2, Oasl1, Pdgfb, Pdk4, Pdlim7, Plat, Plaur, Plin2, Plk3, Plp2, Ppm1j, Ppp1r14b, Pprc1, Procr, Prrg4, Ptger4, Ptgs2, Ptpn12, Pvr, Pxdc1, Qsox1, Rab31, Rad18, Rap2b, Rbm3, Rell1, Rhbdf2, Rin1, Rnd1, Rnd3, Rras2, Rtn4, Runx1, Samd4, Sbno2, Sema7a, Serpinb1a, Serpine1, Sfn, Slc16a1, Slc25a24, Slc38a2, Slc7a5, Slc7a6, Smad1, Smox, Socs3, Sox9, Sphk1, Sprr1a, Sprr2f, Sprr2g, Spry2, Spsb1, St3gal1, Stil, Syt12, Taf1d, Taf4b, Tcerg1, Tgfbr1, Thbd, Timp1, Tinagl1, Tmcc3, Tmed5, Tmem173, Tnc, Tnfrsf12a, Tnfrsf1a, Tpm3, Trmt61a, Tubb6, Txnrd1, Uchl1, Vopp1 |
| Down-regulated  (88 genes) | 1700040L02Rik, Abhd14b, Acad12, Acmsd, Acot11, Acox2, Acss2, Ak4, Akr1c14, Akr1c18, Aldh4a1, Apeh, Atp6v1b1, BC067074, Bcat1, Bdh1, Bhmt2, Bphl, Bsnd, Casr, Cbs, Cdkl1, Ceacam2, Cmah, Crot, Cth, Cubn, Cyp2j11, D3Ertd751e, Dhdh, Dnajc28, Fam107a, Fggy, Fmo5, Fras1, G6pc, Galm, Gatb, Gatm, Glyctk, Gm10804, Hgd, Hnmt, Hsd3b2, Hykk, Ift122, Inpp5j, Kcnj1, Keg1, Klhl3, Kmo, Lrrc31, Lyplal1, Map2k6, Mccc1, Mccc2, Mep1b, Mgam, Nccrp1, Oxgr1, Pbld1, Pde4c, Pfkm, Pnkd, Pter, Ranbp3l, Rhcg, Serpina1d, Serpina1f, Sfrp1, Shmt2, Slc15a2, Slc16a9, Slc22a13, Slc22a19, Slc22a26, Slc23a1, Slc25a21, Slc8a1, Slco4c1, Smarca2, Snx29, Suox, Tln2, Tmem207, Tmem25, Tpmt, Ugt8a |

AKI Acute Kidney Injury, DEGs Differentially Expressed Genes
