# Supplementary material for: Identification and Validation of Potential Biomarkers and Their Functions in Acute Kidney Injury
Source: Front Genet. 2020 May 12;11:411. doi: 10.3389/fgene.2020.00411 (PMC7247857; doi:10.3389/fgene.2020.00411)
Supplement: TABLE S4 — The KEGG pathway of five significant modules selected by MCODE. [file Table_4.doc]

**Table S4 The KEGG pathway of five significant modules selected by MCODE**

| **Module and genes** | **KEGG pathway** | **False discovery rate** | **Gene count** | **Genes** |
| --- | --- | --- | --- | --- |
| Module1:Srgn, Tgfb1, Fgg, Fga, Qsox1, Pdgfb, Clu, Timp1, Tmsb4x, Pros1 | mmu4610: Complement and coagulation cascades | 0.00121 | 3 | Fga,Fgg,Pros1 |
|  | mmu5211: Renal cell carcinoma | 0.0494 | 2 | Pdgfb,Tgfb1 |
| Module2: Casr, F2rl1, Anxa1, F2r, Cxcl12, Gna14, Oxgr1, Cxcl1, Cxcl2, Edn1, Apln | mmu4668: TNF signaling pathway | 0.00479 | 3 | Cxcl1,Cxcl2,Edn1 |
|  | mmu4062: Chemokine signaling pathway | 0.0105 | 3 | Cxcl1,Cxcl12,Cxcl2 |
|  | mmu4060: Cytokine-cytokine receptor interaction | 0.0183 | 3 | Cxcl1,Cxcl12,Cxcl2 |
|  | mmu4064: NF-kappa B signaling pathway | 0.034 | 2 | Cxcl12,Cxcl2 |
| Module3: Nop58, Rrp1b, Sdad1, Ddx21, Gnl3, Aatf | mmu3008: Ribosome biogenesis in eukaryotes | 0.0481 | 2 | Gnl3,Nop58 |
| Module4: Amt, Dmgdh, Mthfd1, Shmt2, Mthfd1l, Mthfd2 | mmu670: One carbon pool by folate | 3.97E-13 | 5 | Amt,Mthfd1,Mthfd1l,Mthfd2,Shmt2 |
|  | mmu1100: Metabolic pathways | 0.00000333 | 6 | Amt,Dmgdh,Mthfd1,Mthfd1l,Mthfd2,Shmt2 |
|  | mmu260: Glycine, serine and threonine metabolism | 0.00000888 | 3 | Amt,Dmgdh,Shmt2 |
|  | mmu1200: Carbon metabolism | 0.0232 | 2 | Amt,Shmt2 |
| Module5: Mut, Acox2, Pcca, Hibch, Aox3, Acss3, Acss2, Eci3, Abat, Acadsb, Mccc2 | mmu280: Valine, leucine and isoleucine degradation | 1.53E-14 | 7 | Abat,Acadsb,Aox3,Hibch,Mccc2,Mut,Pcca |
|  | mmu640: Propanoate metabolism | 2.55E-13 | 6 | Abat,Acss2,Acss3,Hibch,Mut,Pcca |
|  | mmu1100: Metabolic pathways | 1.87E-10 | 10 | Abat,Acadsb,Acox2,Acss2,Acss3,Aox3,Hibch,Mccc2,Mut,Pcca |
|  | mmu1200: Carbon metabolism | 0.0000107 | 4 | Acss2,Hibch,Mut,Pcca |
|  | mmu630: Glyoxylate and dicarboxylate metabolism | 0.00304 | 2 | Mut,Pcca |
|  | mmu1120: Microbial metabolism in diverse environments | 0.00304 | 3 | Acss2,Mut,Pcca |
|  | mmu410: beta-Alanine metabolism | 0.00401 | 2 | Abat,Hibch |
